# Experienced and Anticipated Discrimination and Social Functioning in Persons With Mental Disabilities in Kenya: Implications for Employment

**DOI:** 10.3389/fpsyt.2019.00181

**Published:** 2019-04-05

**Authors:** Ikenna D. Ebuenyi, Barbara J. Regeer, David M. Ndetei, Joske F. G. Bunders-Aelen, Mònica Guxens

**Affiliations:** ^1^Athena Institute, Amsterdam Public Health Research Institute, Vrije Universiteit Amsterdam, Amsterdam, Netherlands; ^2^ISGlobal, Hospital Clínic - Universitat de Barcelona, Barcelona, Spain; ^3^Department of Experimental and Health Sciences, Pompeu Fabra University, Barcelona, Spain; ^4^Department of Psychiatry, University of Nairobi, Nairobi, Kenya; ^5^Africa Mental Health Foundation, Nairobi, Kenya; ^6^Spanish Consortium for Research on Epidemiology and Public Health (CIBERESP), Instituto de Salud Carlos III, Madrid, Spain; ^7^Department of Child and Adolescent Psychiatry/Psychology, Erasmus University Medical Centre–Sophia Children's Hospital, Rotterdam, Netherlands

**Keywords:** mental disability, discrimination, social function, employment, Kenya

## Abstract

**Introduction:** Persons with mental illness experience social life restriction and stigma that may have implications for their work ability. The aims of this study are (i) to report experienced and anticipated discrimination and social functioning in persons with mental disabilities in Kenya and (ii) to investigate the association between experienced and anticipated discrimination, social functioning, and employment in this population.

**Materials and Methods:** Cross-sectional study design where we randomly recruited 72 persons with mental illness through two networks of persons with psychosocial disabilities in Kenya. Experienced and anticipated discrimination were measured using the Discrimination and Stigma Scale version 12 (DISC 12) while social functioning was measured using the Social Functioning questionnaire (SFQ).

**Results:** Experienced discrimination was reported by 81.9% in making or keeping friends, 69.7 and 56.3% in finding or keeping job, respectively, and 63.3% in dating or having an intimate relationship. Anticipated discrimination stopped 59.2% from applying for work, 40.8% from applying for education or training courses, and 63.4% from having a close personal relationship. Females reported an overall higher experienced discrimination than males. Unemployed participants had slightly increased rates of experienced and anticipated discrimination (9.5 vs. 9.1 and 2.5 vs. 2.3, respectively) (*p* > 0.05), while there was a significant association between impaired social functioning and unemployment [14.0 vs. 11.2 (*p* = 0.037)].

**Conclusion:** The rates of experienced and anticipated discrimination faced by persons with mental disabilities in Kenya is high and with significant gender disparity. Although no strong associations were observed between experienced and anticipated discrimination and employment, impaired social functioning of persons with mental disabilities seems to have implications for employment. Further research is essential to understand the predictors of the discrimination and measures to reduce them in persons with psychosocial disabilities.

## Introduction

Globally, mental illness is among the leading causes of disability and social exclusion (1). Persons with mental illness experience social life restriction and stigma that may have implications for their work ability (2, 3). While it is often challenging to untangle the causal links between social functioning, stigma, and the occupational life of persons with mental illness, studies demonstrated that persons with mental illness have increased rates of stigma, impaired social functioning, and unemployment compared to the general population (2–5). These disadvantages have implications for their social participation and human rights. Addressing this imbalance is important but it is still a neglected societal issue especially in low-income countries with paucity of research on mental illness (6, 7).

Studies in high-income countries have demonstrated that stigma for mental illness is manifested through both overt and covert actions that result in discrimination against persons with mental illness (8, 9). These systematic societal attitudes isolate persons with mental illness and produce social disadvantages in major areas of life such as work and school. Experienced discrimination is as a result of perceived unfair treatment while anticipated discrimination occurs when an individual limits his or her activities on account of fear of discrimination (10). A mixed method study by Thornicroft and colleagues that analyzed data from 27 countries revealed that experienced and anticipated discrimination affected the work, education, and social life of persons with mental illness (3). A more recent cross sectional study in the UK that explored coping mechanisms in mental health service users showed that illness concealment as a coping mechanism found in 73% of participants was associated with anticipated discrimination (11). This finding is consistent with a similar study in Australia that reported a 50% rate of both experienced and anticipated discrimination in participants with severe mental illness (12). In Nigeria, a study by Oshodi et al reported that experienced and anticipated discrimination in young people affected their social interactions and work ability (13). Furthermore, studies indicated a gendered pattern to discrimination, with women having higher rates of anticipated discrimination than men (14, 15). Finally, impaired social functioning has also been associated with a lower employability among individuals with mental illness mainly in high income countries (4, 16, 17).

In spite of the abundance of studies linking stigma, impaired social function, and employment in persons with mental disability, few exist in Africa (18). While the evidence in high income countries is growing, it is essential to replicate such studies in low income countries where there is heightened stigma for mental illness. These studies would provide information on the magnitude of the problem in such regions and serve as evidence with which to engage policy makers on the need for the establishment of change processes to mitigate the challenges persons with mental disability face. Therefore, the aims of this study are (i) to report experienced and anticipated discrimination and social functioning in persons with mental disabilities in Kenya and (ii) to investigate the association between experienced and anticipated discrimination, social functioning, and employment in this population.

## Materials and Methods

### Study Design and Population

A cross-sectional study design was employed, where we randomly recruited persons with mental illness through two networks of persons with psychosocial disabilities: Users and survivors of psychiatry (USP) and African Mental Health Foundation (AMHF) in Kenya. The target population was living in Nairobi county and the surrounding rural settlements. A total of 120 persons were invited, and 72 (60%) accepted to participate in the study. Participants answered a researcher designed questionnaire in English or Swahili language, the official languages in Kenya.

### Experienced and Anticipated Discrimination

We used the Discrimination and Stigma Scale version 12 (DISC-12), a 34 item interview-based and standardized tool for assessment of discrimination that has been used in both high income countries and low-and-middle-income countries (3, 10). The DISC-12 has good psychometric properties including inter-rater reliability (weighted kappa range: 0.62–0.95), internal consistency (α = 0.78) and test-retest reliability (weighted kappa range: 0.56–0.89) (10). It consists of a global scale and four subscales: (1) Unfair treatment (item 1–22); (2) Stopping self (item 23–26); (3) Overcoming stigma (item 27–28), and (4) Positive treatment (item 29–34). The unfair treatment subscale assesses unjust treatment by other people and higher scores indicate greater experienced discrimination. The stopping self subscale explores the extent to which an individual limits his/her activities of daily living (e.g., work) due to fear of stigma and higher scores mean higher anticipated discrimination. The overcoming stigma subscale measures an individual's ability to overcome stigma and higher scores indicate a higher ability to cope with discrimination. The positive treatment subscale assesses positive treatment received by an individual on account of mental illness and higher scores mean greater positive treatment received by the individual. The responses to the DISC-12 are rated on a four point Likert scale (Not at all = 0, A little = 1, Moderately = 2, and A lot = 3). The mean for the overall and subscales scores were calculated by summation of the rating (0–3) for each item and dividing with the total number of applicable terms. The count for the total score for the overall and each subscale were calculated by counting the number of items that the individual scored as 1 (a little), 2 (moderately), or 3 (a lot) (19). The higher the scores, the greater the stigma.

### Social Functioning

We used the Social Functioning Questionnaire (SFQ), an eight-item self-reported scale (score range 0–24) that provides a quick assessment of perceived social functioning. It was developed from the Social Functioning Schedule (SFS) and has good test-retest and inter-rater reliability, including construct validity (20, 21). The SFQ are sets of questions that cover diverse life domains such as work, home, relationship, financial problems, sexual life, and relationship (Supplementary Table 1). The responses are on a four point non-uniform scale. A score of 10 or more indicates impaired social functioning (21). We categorized the scale as high (score below 10) and low (score of 10 or above) social functioning.

### Sociodemographic Characteristics and Employment

We obtained information on age, gender, educational level, marital status, number of children, type of mental disability diagnosed, employment status, job satisfaction (among the employed), interest to be employed, and belief on how employment can have an impact on their medical recovery through a self-reported questionnaire.

### Data Management and Statistical Analysis

Descriptive statistics were used to examine the relationship between sociodemographic characteristics and employability by means of means and standard deviations for continuous variables and proportions for categorical variables. Independent samples *t*-test, One way analysis of variance (ANOVA), Chi-square and Fischer's exact test were used to identify group differences between the employed and unemployed depending on the distribution of the independent variables. All analyses were conducted using IBM SPSS version 23 (IBM, New York USA).

## Results

### Participants Characteristics

Socio-demographic characteristics of the participants are shown in Table 1. Out of the 72 participants, 69.4% were females and most of them were unmarried (70.8%). In terms of the self-reported mental illness typology, 31.3% indicated having depression, 29.7% depression together with other comorbid conditions, 20.3% bipolar disorder, and 18.8% schizophrenia and other psychotic conditions. Slightly more than half (55.6%) were unemployed and of those that were employed, half were self-employed and 46.9% were satisfied with their jobs. Overall, a total of 76.8% were interested to be employed.

**Table 1:** Socio-demographic characteristics of the participants (*N* = 72).

**Variable**	**Categories**	**Distribution *N* (%)**
Age	30 years and below	10 (13.9)
	31-40 years	29 (40.3)
	41 years and above	33 (45.8)
Gender	Male	22 (30.6)
	Female	50 (69.4)
Marital status	Unmarried	51 (70.8)
	Married	21 (29.2)
Number of children	None	16 (23.5)
	With children	52 (76.5)
	Missing	*4*
Education level	Primary and below	32 (45.1)
	Secondary and above	39 (54.9)
	Missing	*1*
Type of mental disability diagnosed	Schizophrenia and other psychotic disorders	12 (18.8)
	Depression only	20 (31.3)
	Depression and other comorbid conditions	19 (29.7)
	Bipolar disorder	13 (20.3)
	Missing	*8*
Employment status	Unemployed	40 (55.6)
	Employed^*^	32 (44.4)
Job satisfaction (among the employed)	Satisfied	15 (46.9)
	Not satisfied	17 (53.1)
Interested to be employed	Yes	53 (76.8)
	No	16 (23.2)
	Missing	*3*

* *15 out of the 32 employed participants were self-employed. Italic values indicates participant responses ‘missing’ from each given demographic question*.

### Experienced and Anticipated Discrimination

**Figure 1:**
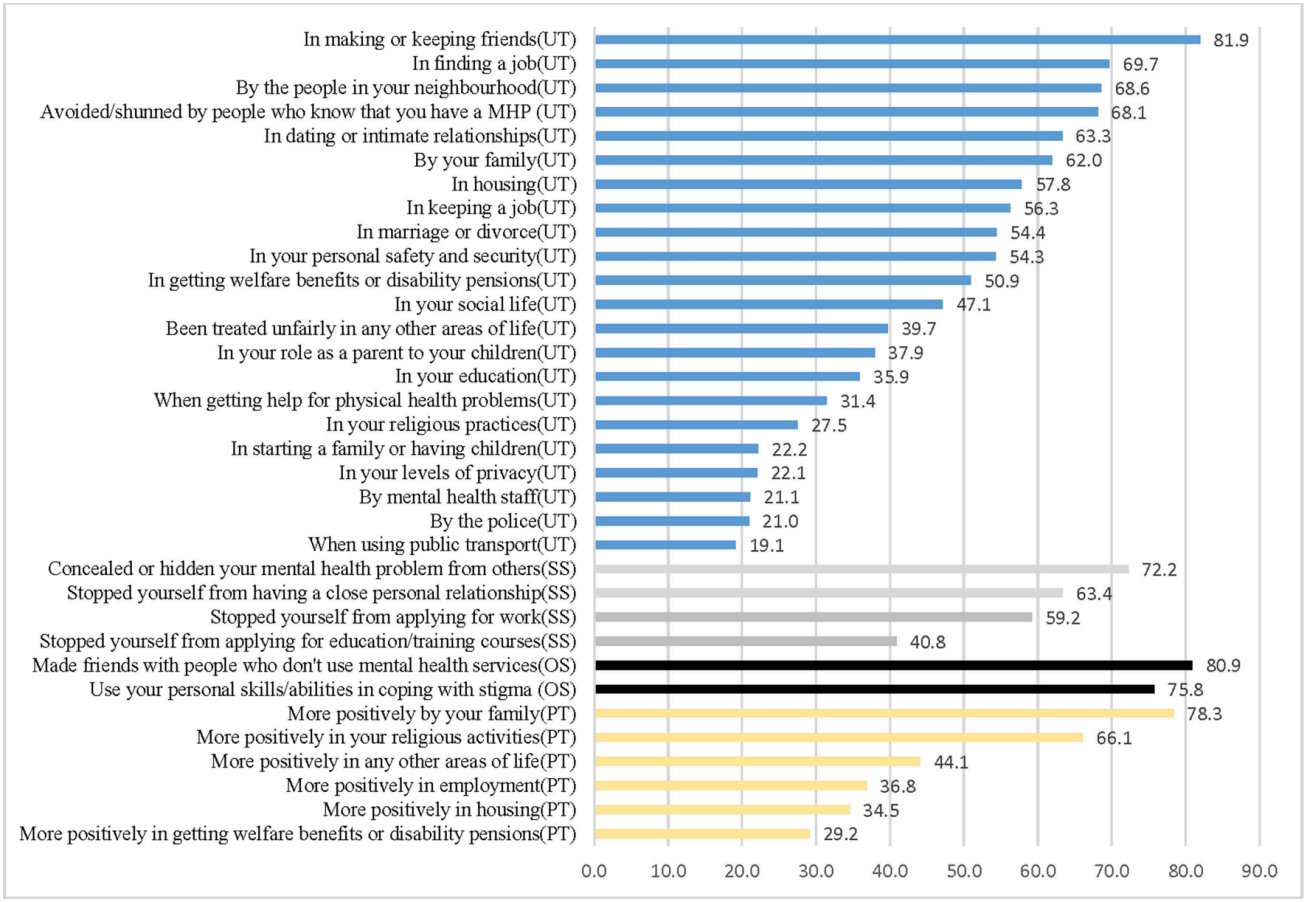
Proportion of agree responses for DISC item. UT, Unfair treatment subscale; SS, Stopping self subscale; OS, Overcoming stigma subscale; PT, Positive treatment subscale; MHP, Mental health problem.

Mean score for experienced discrimination (unfair treatment subscale) was 0.9 (SD = 0.5) and for anticipated discrimination subscale (stopping self subscale) was 1.4 (SD = 0.9) (Table 2). Experienced discrimination (unfair treatment subscale) was reported by 81.9% in making or keeping friends, 69.7 and 56.3% in finding or keeping a job, respectively, and 63.3% in dating or having an intimate relationship (Figure 1, Supplementary Table 2). Anticipated discrimination (stopping self-subscale) stopped 59.2% from applying for work, 40.8% from applying for education or training courses, and 63.4% from having a close personal relationship.

**Table 2:** Stigma and social function scores.

	**Mean (*SD*)**	**Min-Max**.
DISC total mean ^*a*^	1.3 (0.4)	0.4–2.2
Unfair treatment	0.9 (0.5)	0–2
Stopping self	1.4 (0.9)	0–3
Overcoming stigma	1.9 (1.1)	0–3
Positive treatment	0.9 (0.8)	0–3
DISC total count ^*b*^	14.8 (5.4)	0–29
Unfair treatment	9.3 (4.4)	0–18
Stopping self	2.4 (1.2)	0–4
Overcoming stigma	1.5 (0.7)	0–2
Positive treatment	2.4 (2.0)	0–6
Impaired social functioning	**12.8 (5.7)**	**3–23**
Social functioning levels	**High (33.3%)** **Low (66.7%)**	

b *DISC Total Count is the count of the number of items endorsed in the*

a *DISC total scale or subscales; DISC Total Mean is the mean DISC total scale or a subscale score. The bold values are significant values*.

Females reported significantly higher experienced discrimination (unfair treatment subscale) in finding and keeping a job, in housing, and in their personal safety and security while men experienced more discrimination in being shunned or avoided by people who know that they have a mental health problem, in their education, and by the police (Table 3, Supplementary Figure 1).

**Table 3:** Proportion of agree responses for DISC items by gender.

***Have you……*.**	**Males (*N* = 22)**	**Females (*N* = 50)**	***P*-value**
**Unfair treatment Sub-scale**	**(%)**	**(%)**	***P*****-value**
Been treated unfairly In making or keeping friends	81.8	82.0	0.985
**Been treated unfairly by the people in your neighborhood**	50.0	74.0	0.047
Been treated unfairly In dating or intimate relationships	59.1	50.0	0.477
**Been treated unfairly In housing**	19.0	66.0	<0.001
Been treated unfairly In your education	40.9	28.0	0.279
Been treated unfairly In marriage or divorce	31.8	48.0	0.201
Been treated unfairly By your family	52.4	66.0	0.281
**Been treated unfairly In finding a job**	36.4	76.0	0.001
**Been treated unfairly In keeping a job**	31.8	58.0	0.041
Been treated unfairly When using public transport	18.2	18.0	0.985
Been treated unfairly In getting welfare benefits or disability pensions	25.0	47.9	0.080
Been treated unfairly In your religious practices	19.0	30.6	0.319
Been treated unfairly In your social life	42.9	49.0	0.638
Been treated unfairly By the police	27.3	14.3	0.191
Been treated unfairly When getting help for physical health problems	22.7	34.7	0.313
Been treated unfairly By mental health staff	22.7	20.4	0.825
Been treated unfairly In your levels of privacy	18.2	22.4	0.684
**Been treated unfairly In your personal safety and security**	31.8	63.3	0.014
Been treated unfairly In starting a family or having children	15.0	22.0	0.508
Been treated unfairly In your role as a parent to your children	19.0	36.7	0.144
Been avoided or shunned by people who know that you have a mental health problem	81.0	60.0	0.089
Been treated unfairly in any other areas of life	30.0	42.0	0.351
**STOPPING SELF SUB-SCALE**			
**Stopped yourself from applying for work**	33.3	70.0	0.004
Stopped yourself from applying for education or training courses	28.6	46.0	0.173
**Stopped yourself from having a close personal relationship**	33.3	76.0	0.001
Concealed or hidden your mental health problem from others	68.2	74.0	0.612
**OVERCOMING STIGMA SUB-SCALE**			
Made friends with people who don't use mental health services	90.0	77.1	0.217
Been able to Use your personal skills or abilities in coping with stigma and discrimination	75.0	72.9	0.859
**POSITIVE TREATMENT SUB-SCALE**			
Been treated More positively by your family	80.0	64.6	0.210
Been treated More positively in getting welfare benefits or disability pensions	15.0	23.4	0.439
Been treated More positively in housing	40.0	22.9	0.153
Been treated More positively in your religious activities	70.0	52.1	0.173
**Been treated More positively in employment**	50.0	23.4	0.032
**Been treated More positively in any other areas of life**	65.0	27.7	0.004

When comparing the distribution of the socio-demographic characteristics across the discrimination subscales, females had a higher mean score of overall experienced discrimination (unfair treatment subscale) as compared to males (10.0 vs. 7.7) (Table 4). Those diagnosed with depression together with other conditions had the highest mean score of overall experienced discrimination (unfair treatment subscale) (mean = 11.9), followed by depression only (mean = 8.4), schizophrenia and other psychotic disorders (mean = 8.8), and bipolar disorder (mean = 7.1). Participants unmarried and with one or more children had a slightly higher mean score of overall experienced discrimination (unfair treatment subscale) compared to those married and without children respectively (9.8 vs. 8.0 and 9.8 vs. 7.8, respectively). Regarding anticipated discrimination (stopping self-subscale), females had a higher overall score as compared to males (2.7 vs. 1.7), as well as unmarried participants compared to those married (2.6 vs. 1.9), and those with secondary or higher educational level compared to those with primary or lower level (2.6 vs. 2.1). No relevant differences were found between socio-demographic characteristics and overcoming stigma besides a slightly higher mean score in participants with secondary or higher educational level compared to those with primary or lower educational level (1.7 vs. 1.4). Males had a higher score in the positive treatment subscale compared to females (3.2 vs. 2.0) and those diagnosed with schizophrenia and psychosis had higher mean score compared to those diagnosed with other mental illness (Table 4).

**Table 4:** Association between socio-demographic characteristics and unfair treatment, stopping self, overcoming stigma, positive treatment, and social functioning.

**Variable**	**Categories**	**Unfair treatment**		**Stopping self**		**Overcoming stigma**		**Positive treatment**		**Social functioning**	
		**Mean (*SD*)**	***P*-value**	**Mean (*SD*)**	***P*-value**	**Mean (*SD*)**	***P*-value**	**Mean (*SD*)**	***P*-value**	**Mean (*SD*)**	***P*-value**
Gender	Male	7.7 (4.4)	**0.053**	1.7 (1.3)	**0.003**	1.7 (0.6)	0.425	3.2 (2.0)	**0.020**	9.6 (4.8)	**0.001**
	Female	10.0 (4.3)		2.7 (1.1)		1.5 (0.7)		2.0 (1.9)		14.1 (5.5)	
Age	30 and below	8.3 (3.9)	0.726	2.2 (1.3)	0.750	1.6 (0.7)	0.887	2.8 (1.8)	0.732	11.0 (4.8)	0.550
	31–40	9.3 (4.9)		2.5 (1.2)		1.6 (0.6)		2.3 (2.3)		13.3 (5.9)	
	41 and above	9.6 (4.2)		2.3 (1.3)		1.5 (0.8)		2.2 (1.8)		12.8 (5.7)	
Marital Status	Unmarried	9.8 (4.5)	0.118	2.6 (1.2)	**0.043**	1.5 (0.7)	0.965	2.3 (1.9)	0.598	13.6 (5.5)	**0.053**
	Married	8.0 (4.1)		1.9 (1.4)		1.6 (0.8)		2.6 (2.1)		10.8 (5.7)	
Number of children	None	7.8 (4.3)	0.118	2.1 (1.4)	0.359	1.7 (0.6)	0.352	2.9 (1.9)	0.263	10.6 (4.1)	**0.047**
	One or more	9.8 (4.4)		2.5 (1.2)		1.5 (0.7)		2.2 (2.0)		13.8 (5.9)	
Education level	Primary and below	9.8 (4.2)	0.444	2.1 (1.3)	0.100	1.4 (0.8)	0.054	2.3 (2.1)	0.787	14.6 (5.7)	**0.016**
	Secondary and above	9.0 (4.7)		2.6 (1.1)		1.7 (0.6)		2.4 (1.9)		11.4 (5.2)	
Diagnosis	Schizophrenia and psychosis	8.8 (3.8)	**0.013**	2.1 (1.2)	0.181	1.5 (0.7)	0.481	3.4 (1.8)	0.139	10.1 (5.6)	**0.004**
	Depression only	8.4 (5.6)		2.1 (1.3)		1.4 (0.7)		1.6 (2.5)		12.6 (5.0)	
	Depression and conditions	11.9 (3.2)		2.7 (1.2)		1.4 (0.8)		2.0 (1.7)		16.0 (5.9)	
	Bipolar disorder	7.1 (3.3)		2.8 (1.0)		1.8 (0.6)		2.3 (1.3)		9.8 (3.9)	
Interested to be Employed	Yes	9.1 (4.3)	0.855	2.3 (1.2)	0.531	1.5 (0.7)	0.298	2.3 (1.9)	0.926	12.9 (5.8)	0.943
	No	9.4 (4.5)		2.6 (1.3)		1.7 (0.6)		2.2 (2.0)		12.8 (5.7)	

*The bold values are significant values*.

### Social Functioning

Mean social functioning score was 12.8 (SD = 5.7) and about 2/3 of the participants had low social functioning (Table 2). As shown in Table 4, females had higher impaired social function scores as compared to males (14.1 vs. 9.6) as well as those unmarried compared to those who were married (13.6 vs. 10.8). Participants with children had higher impaired social functioning as compared to those without children (13.8 vs. 10.6), as well as participants with primary level of education and below as compared to those with secondary and above level of education (14.6 vs. 11.4). Those diagnosed with depression together with other conditions had the highest score of impaired social functioning (mean = 16.0), followed by depression only (mean = 12.6), schizophrenia and other psychotic disorders (mean = 10.1), and bipolar disorder (mean = 9.8).

### Associations Between Socio-Demographic Characteristics, Experienced and Anticipated Discrimination, Social Functioning, and Employment

The age of the participants was significantly different between unemployed and employed, where those who were younger were more likely to be unemployed as compared to those who were older (Table 5). Females and those without children were slightly more likely to be unemployed than males and those with children, respectively.

**Table 5:** Factors associated with employability.

**Variable**	**Categories**	**Unemployed** ***N* = 40**	**Employed** ***N* = 32**	***P*-value**
Age	30 years and below	22.9	3.1	**0.014**
	31–40 years	45.2	34.4	
	41 years and above	32.5	62.5	
Gender	Male	22.5	40.6	0.097
	Female	77.5	59.4	
Marital status	Unmarried	75.0	65.6	0.384
	Married	25.0	34.4	
Children	None	30.8	13.8	0.103
	With children	69.2	86.2	
Education level	Primary and below	50.0	38.7	0.343
	Secondary and above	50.0	61.3	
Type of mental disability	Schizophrenia and other psychotic disorders	11.4	27.6	0.341
	Depression only	37.1	24.1	
	Depression and other comorbid conditions	28.6	31.0	
	Bipolar disorder	22.9	17.2	
Interested to be employed	Yes	81.6	71.0	0.299
	No	18.4	29.0	
Think that employment has/would have an impact on your medical outcome/recovery	Yes	86.8	93.5	0.446
	No	13.2	6.5	
Unfair Treatment		9.5 (4.6)	9.1 (4.2)	0.698
Stopping Self		2.5 (1.3)	2.3 (1.2)	0.448
Overcoming Stigma		1.5 (0.7)	1.7 (0.7)	0.263
Positive Treatment		2.3 (2.1)	2.4 (1.8)	0.955
Impaired social functioning		14.0 (5.1)	11.2 (6.0)	**0.037**
Social functioning levels	High	22.5	46.9	**0.029**
	Low	77.5	53.1	

*Values are percentages for categorical variables and mean (SD) for continuous variables. The bold values are significant values*.

Although participants who were unemployed reported slightly higher scores of experienced and anticipated discrimination (unfair treatment and stopping self subscales) (9.5 vs. 9.1 and 2.5 vs. 2.3, respectively), no significant association was found between discrimination and unemployment. However, there was an association between impaired social function and employment status. Those who were unemployed had higher impaired social functioning than those who were employed [14.0 vs. 11.2 (*p* = 0.037)].

## Discussion

Our study, one of the few carried out in Africa, showed elevated rates of experienced discrimination among people with mental disabilities, particularly in finding and keeping jobs. Similarly, anticipated discrimination stopped the majority of the participants from applying for work or education. Female participants experienced higher discrimination in finding and keeping a job and accessing education than males, as well as in all the assessed domains of anticipated discrimination including work and education. Those participants who were unemployed had only slightly higher rates of experienced and anticipated discrimination. However, we found increased rates of impaired social function among people with mental disabilities and this was significantly higher in those who were unemployed.

Our study recorded a higher rate of experienced discrimination than the one reported by Thornicroft and colleagues in their multi-country study on discrimination (69.7 vs. 29%) (3). Overall mean scores of experienced and anticipated discrimination in our study were also higher than those reported in a recent cross sectional study from China (0.9 and 1.4 in our study vs. 0.20 and 0.79 in the study from China, respectively) (22). These increased rates are rather worrisome and perhaps not surprising on account of the cultural stereotypes surrounding mental illness in Kenya (23) and in most low income countries (6). In Kenya, the traditional perception is that persons with mental illness are mad, insane, violent and likely to harm themselves and others (23). Our findings highlight the need for interventions in order to reduce stigma toward people with mental disabilities in Kenya, as well as in similar low income countries, and to mitigate the negative social and life implications that stigma has on these people. In line with that, there is already some recent evidence from a pilot study in Kenya that demonstrated the usefulness of an intervention, following the World Health Organization mental health Gap action Programme guide, in the reduction of experienced discrimination by persons with mental disorders (5). Further research and interventions are needed in particular in low income countries.

Another important and worrisome finding in our study was the gender pattern of stigma. Females reported higher rates of experienced and anticipated discrimination in work, education, and social life. This finding was corroborated by a study in Pakistan where women had higher rates of internalized stigma than men (24). Similarly, two different studies from India (15) and the UK (14) reported higher rates of anticipated discrimination in women. Conversely, a Spanish cross-sectional study showed that men reported more anticipated discrimination than women (25). In contrast, no gender differences were found in anticipated discrimination in the multi-country study by Thornicroft and colleagues (3) and in a cross-sectional study from Nigeria (13). The different findings between studies regarding the gender differences in reported anticipated discrimination and experienced discrimination may be related to several factors specific to socio-cultural factors (e.g., gender roles and local beliefs and practices) in the setting and the illness specific factors. A study from the US including African Americans participants found that age and gender differences were reported in attitude, perception, and adopted (religious) coping mechanisms against mental illness stigma (26).

Although we did not find big differences between discrimination and unemployment in our study, the observed slightly higher rates of experienced and anticipated discrimination in those who were unemployed were supported by findings from two multi-country studies (2, 3) where stigma was identified as a barrier to social and vocational integration. Previous studies also documented a relationship between mental illness, stigma, and unemployment, and its implication on the lives of affected individuals (4, 8). It is possible the experienced and anticipated discrimination were solely on account of unemployment and not mediated by mental illness. However, we did not explore these relationship in our study. Employment for persons with mental disability is a human right and also important for their recovery and social participation (27). As discrimination against persons with mental illness has been shown to affect work ability and opportunities, the United Nations Convention on the Rights of Persons with Disabilities (UNCRPD) advocates for equality in employment (28). Studies in high income countries pointed to disparities in employment opportunities between persons with mental disability and the general population (29). Also important is the finding by Lasalvia and colleagues who observed that experienced discrimination was associated with reduced willingness to disclose ones diagnosis with depression (2), which might work against securing reasonable accommodation in employment (30). Similarly to this previous study, our study participants might also be unwilling to disclose their mental illness on account of the heightened stigma against mental illness in the setting.

We noted that about 2/3 of our study participants had impaired social function and that those who were unemployed were more likely to have impaired social function than those who were employed. The association between impaired social function and mental illness is common and had previously been documented (21, 31). However, our study reveals its implication for employment and the well-being of affected individuals for the first time in an African country. Also interesting is the fact that our study found impaired social function in those with primary level of education and below compared to those with secondary level of education. This finding strengthens the pivotal relationship between education and employment, especially in low income countries where higher or more education is essential for employability (32). It is pertinent to note that individuals with depression and other comorbid illness (e.g., substance use) had higher rates of experienced discrimination and impaired social function scores in our study. This may be due to synergistic effect of syndemics and calls for greater care for affected individuals on account of the impact of the multiple disadvantages on their employment opportunities.

Our study is not without some glimmer of hope. Participants reported being treated more positively by family and in religious activities. This is rather encouraging as two different studies reported that positive experienced discrimination is rare (3, 33). The importance of this finding is that family and religious organizations may serve as a contact point for interventions for stigma reduction in persons with mental disabilities. This suggestion conflates with the recommended partnership between faith based organizations and mental health services for the well-being of person with mental disabilities (26).

The main strength of our study is related to its novelty and being the first in Kenya and to the best of our knowledge in East Africa. Our exploratory study set out to draw attention to this neglected group and the barriers of social exclusion they endure. However, our study is limited by the use of a modest sample size, which might have been underpowered to detect stronger associations between discrimination and unemployment. Also, our reliance on self-reported questionnaires may have been affected by memory or recall bias. Thus, responses may have been overestimated or underestimated; and may not completely reflect the actual experiences of the individuals. It is possible that the experienced and anticipated discrimination reported by the study participants were on account of double stigma from both mental illness and unemployment (34). However, we were unable to disentangle between these two sources of stigma. Lastly, it is pertinent to note that the first two items of the social function questionnaire are directly related to work and may have affected the assessment of the association between social function and employment in this study.

## Conclusions

The rates of experienced and anticipated discrimination faced by persons with mental disabilities in Kenya is high. The gendered disparity in anticipated and experienced discrimination in persons with mental disability in Kenya may indicate the disadvantages faced by women with mental disabilities in traditional African societies. Although no strong association was observed between experienced and anticipated discrimination and unemployment, impaired social function of persons with mental disabilities seems to have implications for employment. Further longitudinal and intervention studies are essential to understand the relationship between discrimination, social dysfunction, and mental illness, as well as measures that might be useful for improving work life of persons with mental disabilities in particular in low income countries.

## Ethics Statement

The study design was approved by the Amsterdam Public Health science committee (WC2017-011). The Maseno University Ethics Review committee approved the study (MSU/DRPI/MUERC/00391/17). Maseno University Ethics Review Committee (MUERC) is the Institutional Review Board (IRB) of Maseno University and has a mandate from the National Commission for Science, Technology and Innovation (NACOSTI) Kenya to grant review and grant approvals for research in Kenya. Informed consent was obtained from all study participants.

## Author Contributions

IE, BR, and JB-A were involved in the research design. IE collected the data, analyzed the data, and wrote the initial draft with MG. IE, BR, DN, JB-A, and MG revised the manuscript. All authors approved the final version of the manuscript for submission.

### Conflict of Interest Statement

The authors declare that the research was conducted in the absence of any commercial or financial relationships that could be construed as a potential conflict of interest.
