# Erratum: Experienced and Anticipated Discrimination and Social Functioning in Persons With Mental Disabilities in Kenya: Implications for Employment

**DOI:** 10.3389/fpsyt.2021.726872

**Published:** 2021-07-08

**Authors:** Frontiers Production Office

**Affiliations:** Frontiers Media SA, Lausanne, Switzerland

**Keywords:** mental disability, discrimination, social function, employment, Kenya

Due to a production error, the incorrect Supplementary Materials files were uploaded.

The publisher apologizes for this mistake. The original article has been updated.

