# Supplementary material for: Experienced and Anticipated Discrimination and Social Functioning in Persons With Mental Disabilities in Kenya: Implications for Employment
Source: Front Psychiatry. 2019 Apr 5;10:181. doi: 10.3389/fpsyt.2019.00181 (PMC6459950; doi:10.3389/fpsyt.2019.00181)
Supplement: Supplementary file 1 [file Data_Sheet_1.docx]

**Supplementary Appendix**

Experienced and anticipated discrimination and social functioning in persons with mental disabilities in Kenya: implications for employment

Ikenna D. Ebuenyi, Barbara J. Regeer, David M. Ndetei, Joske F. G. Bunders-Aelen, and Mònica Guxens

Table of Contents

[Supplementary Table 1. Percentage of respondents scoring on each item of SFQ items 2](#_Toc518648674)

[Supplementary Table 2. Responses to individual DISC items 3](#_Toc518648675)

[Supplementary Figure 1. Negative Discrimination item percentages by gender. Note-UT-unfair treatment; MHP-Mental health Problem. 4](#_Toc518648676)

## Supplementary Table 1. Percentage of respondents scoring on each item of SFQ items

| **Question** | **Response** | **%** |
| --- | --- | --- |
| I complete my tasks at work and home satisfactorily | Most of the time | 27.8 |
|  | Quite often | 16.7 |
|  | Sometimes | 52.8 |
|  | Not at all | 2.8 |
| I find my task at work and at home very stressful. | Most of the time | 31.9 |
|  | Quite often | 11.1 |
|  | Sometimes | 47.2 |
|  | Not at all | 9.7 |
| I have no money problems. | No problems at all | 2.8 |
|  | Slight worries only | 18.1 |
|  | Definite problems | 29.2 |
|  | Very severe problems | 50.0 |
| I have difficulties in getting and keeping close Relationships. | Severe difficulties | 30.6 |
|  | Some problems | 20.8 |
|  | Occasional problems | 26.4 |
|  | No problems at all | 22.2 |
| I have problems with my sex life. | Severe problems | 29.2 |
|  | Moderate problems | 23.6 |
|  | Occasional problems | 23.6 |
|  | No problems at all | 22.2 |
| I get on well with my family and other relatives | Yes, definitely | 26.4 |
|  | Yes, usually | 13.9 |
|  | No, some problems | 44.4 |
|  | No, severe problems | 15.3 |
| I feel lonely and isolated from other people. | Almost all the time | 26.4 |
|  | Much of the time | 23.6 |
|  | Not usually | 30.6 |
|  | Not at all | 19.4 |
| I enjoy my spare time. | Very much | 36.1 |
|  | Sometimes | 26.4 |
|  | Not often | 8.3 |
|  | Not at all | 29.2 |

## Supplementary Table 2. Responses to individual DISC items

|  | **Not at all** | **A little** | **Moderately** | **A lot** | (%) |
| --- | --- | --- | --- | --- | --- |
| **Unfair treatment subscale** |  |  |  |  |  |
| Have you been treated unfairly in making or keeping friends | 18.1 | 25.0 | 16.7 | 40.3 | 81.9 |
| Have you been treated unfairly by the people in your neighborhood | 31.4 | 14.3 | 14.3 | 40.0 | 68.6 |
| Have you been treated unfairly in dating or intimate relationships | 36.7 | 18.3 | 13.3 | 31.7 | 63.3 |
| Have you been treated unfairly in housing | 42.2 | 6.3 | 9.4 | 42.2 | 57.8 |
| Have you been treated unfairly in your education | 64.1 | 12.5 | 3.1 | 20.3 | 35.9 |
| Have you been treated unfairly in marriage or divorce | 45.6 | 7.0 | 7.0 | 40.4 | 54.4 |
| Have you been treated unfairly by your family | 38.0 | 21.1 | 14.1 | 26.8 | 62.0 |
| Have you been treated unfairly in finding a job | 30.3 | 7.6 | 12.1 | 50.0 | 69.7 |
| Have you been treated unfairly in keeping a job | 43.8 | 14.1 | 15.6 | 26.6 | 56.3 |
| Have you been treated unfairly when using public transport | 80.9 | 10.3 | 5.9 | 2.9 | 19.1 |
| Have you been treated unfairly in getting welfare benefits or disability pensions | 49.1 | 7.3 | 9.1 | 34.5 | 50.9 |
| Have you been treated unfairly in your religious practices | 72.5 | 14.5 | 2.9 | 10.1 | 27.5 |
| Have you been treated unfairly in your social life | 52.9 | 18.6 | 20.0 | 8.6 | 47.1 |
| Have you been treated unfairly by the police | 79.0 | 4.8 | 4.8 | 11.3 | 21.0 |
| Have you been treated unfairly when getting help for physical health problems | 68.6 | 12.9 | 8.6 | 10.0 | 31.4 |
| Have you been treated unfairly by mental health staff | 78.9 | 8.5 | 8.5 | 4.2 | 21.1 |
| Have you been treated unfairly in your levels of privacy | 77.9 | 8.8 | 5.9 | 7.4 | 22.1 |
| Have you been treated unfairly in your personal safety and security | 45.7 | 14.3 | 11.4 | 28.6 | 54.3 |
| Have you been treated unfairly in starting a family or having children | 77.8 | 6.3 | 4.8 | 11.1 | 22.2 |
| Have you been treated unfairly in your role as a parent to your children | 62.1 | 12.1 | 5.2 | 20.7 | 37.9 |
| Have you been avoided or shunned by people who know that you have a mental health problem | 31.9 | 21.7 | 14.5 | 31.9 | 68.1 |
| Have you been treated unfairly in any other areas of life | 60.3 | 16.2 | 10.3 | 13.2 | 39.7 |
| **Stopping self subscale** |  |  |  |  |  |
| Have you stopped yourself from applying for work | 40.8 | 12.7 | 18.3 | 28.2 | 59.2 |
| Have you stopped yourself from applying for education or training courses | 59.2 | 11.3 | 8.5 | 21.1 | 40.8 |
| Have you stopped yourself from having a close personal relationship | 36.6 | 11.3 | 12.7 | 39.4 | 63.4 |
| Have you concealed or hidden your mental health problem from others | 27.8 | 13.9 | 13.9 | 44.4 | 72.2 |
| **Overcoming stigma subscale** |  |  |  |  |  |
| Have you made friends with people who don't use mental health services | 19.1 | 10.3 | 13.2 | 57.4 | 80.9 |
| Have you been able to use your personal skills or abilities in coping with stigma and discrimination | 24.2 | 12.1 | 16.7 | 47.0 | 75.8 |
| **Positive treatment subscale** |  |  |  |  |  |
| Have you been treated more positively by your family | 21.7 | 15.0 | 20.0 | 43.3 | 78.3 |
| Have you been treated more positively in getting welfare benefits or disability pensions | 70.8 | 8.3 | 12.5 | 8.3 | 29.2 |
| Have you been treated more positively in housing | 65.5 | 10.9 | 10.9 | 12.7 | 34.5 |
| Have you been treated more positively in your religious activities | 33.9 | 8.5 | 11.9 | 45.8 | 66.1 |
| Have you been treated more positively in employment | 63.2 | 12.3 | 8.8 | 15.8 | 36.8 |
| Have you been treated more positively in any other areas of life | 55.9 | 8.5 | 18.6 | 16.9 | 44.1 |

## Supplementary Figure 1. Negative Discrimination item percentages by gender. Note-UT-unfair treatment; MHP-Mental health Problem.
